# Factors Associated with Medication Non-Adherence in Patients with Dyslipidemia

**DOI:** 10.3390/healthcare9070813

**Published:** 2021-06-28

**Authors:** Eman Alefishat, Anan S. Jarab, Walid Al-Qerem, Lina Abu-Zaytoun

**Affiliations:** 1 Department of Pharmacology, College of Medicine and Health Science, Khalifa University of Science and Technology, Abu Dhabi 127788, United Arab Emirates; 2 Center for Biotechnology, Khalifa University of Science and Technology, Abu Dhabi 127788, United Arab Emirates; 3 Department of Biopharmaceutics and Clinical Pharmacy, Faculty of Pharmacy, The University of Jordan, Amman 11942, Jordan; 4 Department of Clinical Pharmacy, Faculty of Pharmacy, Jordan University of Science and Technology, Irbid 22110, Jordan; asjarab@just.edu.jo (A.S.J.); linaabuzaiton96@gmail.com (L.A.-Z.); 5 Department of Pharmacy, Faculty of Pharmacy, Al-Zaytoonah University of Jordan, Amman 11733, Jordan; waleed.qirim@zuj.edu.jo

**Keywords:** adherence, dyslipidemia, statin

## Abstract

Lack of medication adherence among patients with dyslipidemia negatively affects health-related outcomes. This study aims to evaluate medication adherence; we also aim to investigate the predictors of non-adherence among patients with dyslipidemia in Jordan. Medication adherence was evaluated in a total of 228 dyslipidemia patients. The Beliefs about Medicines Questionnaire was also used to assess patients’ beliefs about medications. The majority of the current study participants (73.2%) reported non-adherence to the prescribed medications. There were significant negative associations between medication adherence and concerns of prescription drug use (B = −0.41, *p*-value < 0.01), duration of dyslipidemia (B = −0.22, *p*-value < 0.01), and the number of medications (B = −0.64, *p*-value < 0.01). Positive associations were found between medication adherence and the necessity of prescription drug use (B = 0.43, *p*-value < 0.01), taking statin and fibrate (B = 2.04, *p*-value < 0.01), and moderate-intensity statin (B = 2.34, *p*-value < 0.01). As for patients’ beliefs about medications, the item “My medicine to lower my cholesterol disrupted my life” had the highest mean (3.50 ± 0.99). This study revealed a low adherence rate to medication among patients with dyslipidemia. It also demonstrates modifiable factors such as beliefs regarding perceived risk, medication harms, treatment duration, and the number of medications associated with poor adherence in patients with dyslipidemia.

## 1. Introduction

Dyslipidemia, characterized by elevated levels of serum total cholesterol (TC), triglycerides (TG), and low-density lipoprotein cholesterol (LDL-C) in addition to low levels of high-density lipoprotein cholesterol (HDL-C), is one of the major risk factors contributing to cardiovascular disease through atherosclerosis development and progression [1]. Lipoprotein a has also been shown as an independent risk factor for cardiovascular diseases [2]. Among LDL particles, small dense LDL and oxidized LDL have been reported to be the most important contributors to the development of atherosclerotic cardiovascular disease (ASCVD) [3,4]. Earlier studies have shown that controlling LDL-C is a cornerstone in the prevention and treatment of ASCVD [5].

The epidemic of cardiovascular diseases has been observed in developing countries and has become the leading cause of death worldwide [6,7]. In 2015, an estimated 17.7 million people died from ASCVD, representing 31% of international deaths [8]. According to the World Health Organization (WHO), cardiovascular diseases are estimated to account for 37% of all deaths in Jordan in 2017, placing Jordan in 65th place in the world [9]. The prevalence of dyslipidemia among Jordanian adults in 2010 was high, with an average of 48.8% with elevated cholesterol, 40.7% with elevated LDL-C, 43.6% with elevated TG, and 40.1% with reduced HDL-C [10].

It has been shown that medication therapy for dyslipidemia reduces the rate of morbidity and mortality [11,12]. Nevertheless, health-related outcomes among patients with dyslipidemia are suboptimal due to lack of medication adherence [13]. The DA VINCI study, which was conducted on 5888 patients on lipid-lowering therapy from 18 countries across Europe, revealed that less than half of patients with high or very high-risk cardiovascular risk achieved their LDL cholesterol goals [14].

In addition to the challenge of lifestyle modifications, patients with dyslipidemia need to follow complex medication regimens, which could involve multiple medications, with several adverse effects that could negatively affect medication adherence [15,16,17]. Adherence is defined as “the extent to which a person’s behavior in terms of taking the drug and following lifestyle advice is consistent with the recommendations agreed by healthcare providers”[18]. Understanding the factors associated with medication non-adherence can augment efforts to improve it [19]. Although several studies have been conducted to explore variables associated with medication non-adherence in patients with high risk for ASCVD such as hypertension and diabetes in Jordan [20,21], there is no reported date on medication non-adherence and its associated factors among patients with dyslipidemia in Jordan. Therefore, the present study aimed to evaluate medication non-adherence and explore its associated variables among patients with dyslipidemia in Jordan.

## 2. Materials and Methods

**Study Site and Subjects**

In the current cross-sectional study, patients were recruited from the outpatient clinic at the Royal Medical Services Hospital, the University of Jordan Hospital in Amman, and King Abdullah Hospital in Irbid over a period of three months (December 2017–February 2018). Criteria for inclusion of patients were an age of 18 years or older, diagnosis with dyslipidemia for six months or more, and a prescription with at least one drug to control blood lipids. Patients who suffered from cognitive impairment, as documented by their consultant, were excluded from the study. During their outpatient visit to cardiology and internal medicine clinics, patients with biomedical and other laboratory tests measured were evaluated for eligibility to participate in the study. Before interviewing the eligible patients, the research pharmacist collected the socio-demographic and clinical variables at the same time for each patient. A custom-designed questionnaire was used to collect socio-demographic variables, including age, gender, educational level, income, marital status, and smoking behavior, in addition to clinical variables including: type and number of comorbidities, the prescribed medications, duration of dyslipidemia, number and frequency of lipid-lowering medications. Medical charts of eligible patients were used to collect information on systolic (SBP) and diastolic (DBP) blood pressure, TC, LDL-C, HDL-C, TG, and body mass index (BMI). Out of 360 patients who met the inclusion criteria, a total of 228 patients agreed to participate and signed consent. The patients were interviewed in a separate room at the outpatient clinic with an average time of twenty minutes per interview. 

**Sample size calculation**

The following equation was used to compute the minimum sample size required to conduct ordinal regression: 50 + 8P, where p is the number of predictors. The original aim of the study was to evaluate the association of the eighteen variables with the adherence level (Table 1). Therefore, the minimum required sample size was 194 [22].

**Ethics statement**

All participants were familiarized with the study through the study information sheet, and those who agreed to participate were asked to sign a consent form. Participants were informed that their participation in the study is voluntary, they can withdraw from the study at any time, and that this will not affect the service they receive from the hospital. The current study received ethical approval from the institutional review board of the King Abdullah University Hospital (389–2017), the University of Jordan (80/2016/638), and the RMS (TF3/1/PE/11088) in November 2017.

**Study instruments**

**The 4-item medication adherence scale**

The validated Arabic version of the 4-item medication adherence scale was used in the present study [23]. The four items were: Do you forget to take your medications? Are you careless about the time of taking your medications? Do you stop taking your medications when you feel better? Do you stop taking your medications when you feel worse? According to their responses, patients were divided into three groups: patients who reported three or more “yes” responses were considered low adherence, those who reported one or two “yes” responses were deemed to have moderate adherence, while those who reported four “no” answers were considered to have high adherence.

Beliefs about Medicines Questionnaire (BMQ)-specific was translated from English to Arabic and back-translated by two different translators; two versions were compared and found to be comparable. BMQ-specific is a 10-item questionnaire of two scales, of which 5 for each scale evaluate common personal beliefs about the necessity and concerns of prescription drug use [24]. Responses were scored on a Likert scale of five points, where 1 = strongly agree, 2 = agree, 3 = not certain, 4 = disagree, and 5 = strongly disagree. The necessity items were as follows: My medicine to lower my cholesterol protects me from becoming sick; My health depended on medicine to lower my cholesterol; Lowering my cholesterol requires medication; My life would have been impossible without medications to lower my cholesterol; Without medicine to lower my cholesterol, I may have become very ill. Concerns items were as follows: I sometimes worried about becoming too dependent on medicine to lower my cholesterol, My medicine to lower my cholesterol disrupted my life, I sometimes worried about the long-term effects of medicine to lower my cholesterol, Having to take medicine to lower my cholesterol worried me, My medicine to lower my cholesterol was a mystery to me. The necessity and concerns scales had high alpha Cronbach (above 0.8), indicating acceptable internal consistency.

**Statistical analysis**

Data were analyzed using SPSS software version (IBM, Armonk, NY, USA) [25]. Categorical variables were expressed as frequencies and percentages, while continuous variables were expressed as means (SD). Spearman’s rank order correlations were used to evaluate the association between ordinal/interval variables and adherence level (ordinal variable). Kruskal–Wallis one-way analysis of variance was used to assess the differences in adherence level across different levels in categorical variables. A stepwise forward ordinal regression model was conducted to evaluate different variables’ association with adherence level. Model assumptions were reviewed prior to performing the ordinal regression. Multicollinearity was assessed by examining variance inflation factor (VIF) and tolerance values (VIF less than ten and tolerance greater than 0.2), and proportional odds were assessed by examining the parallel line test (*p*-value greater than 0.05). The stepwise ordinal regression model included adherence level as the dependent variable, and the predictors in the model were age, gender, material status, education level, living condition, income level, smoking status necessity, concerns, duration of dyslipidemia (years), presence of different comorbidities, number of medications, and type of medication. A high correlation was found between the number of medications and the number of chronic conditions; therefore, the number of chronic conditions was excluded from the final model.

## 3. Results

A total of 228 (51.8% males) participants were included in the study. About one-third (32.8%) of the participants reported low medication adherence, and only 26.7% reported high adherence. Table 1 represents the association between sample characteristics and adherence level. The high adherent participants had significantly lower total cholesterol, LDL, and total cholesterol/HDL (*p*-values < 0.01). Patients on no or moderate-intensity statins reported higher adherence levels (85% and 40.2%, respectively, *p*-values < 0.01). Among patients with uncontrolled dyslipidemia, 41.7% had low adherence levels, and 40.3% were moderately adherent.

As shown in Table 2, patients generally reported greater “concerns” mean scores than “necessity” scores. The item “My medicine to lower my cholesterol protect me from becoming sick” had the lowest mean (2.32 ± 1.10). In contrast, the item “My medicine to lower my cholesterol disrupted my life” had the highest mean (3.50 ± 0.99).

Table 3 shows the results of stepwise forward ordinal regression. Variables including the increase in medication necessity score, receiving a moderate-intensity statin, receiving statin with fibrates, or not taking statins significantly increased medication adherence (*p*-value < 0.01). On the other hand, a higher medication concerns score, a higher number of medications, and a longer duration of dyslipidemia significantly decreased medication adherence (*p*-values < 0.01).

## 4. Discussion

Despite the availability of effective medications for lipid control, low adherence may limit their benefits [25,26]. Poor adherence to lipid-lowering therapy is associated with adverse cardiovascular effects, increased mortality, and increased healthcare costs [27]. Earlier studies have explored variables associated with medication non-adherence in patients at high risk for ASCVD, such as hypertension [21] and diabetes [20], in Jordan. The present study is the first to evaluate medication adherence and investigate the factors associated with medication non-adherence in patients with dyslipidemia in Jordan.

More than one-third (≈ 33%) of the participants were low adherents, and only 26.7% were high adherents in the present study. The majority of the current study participants (≈ 73%) reported non-adherence to the prescribed medications. The rate of non-adherence reported in the present study is comparable with earlier studies conducted on patients with hypertension (81%) [21], diabetes (72.5%) [20], and angina (79.4%) [28], using the validated Arabic version of the 4-item medication adherence scale in Jordan. The high non-adherence rate reported in this study sheds light on the necessity to develop effective pharmaceutical care intervention programs aimed at improving medication adherence and hence, health outcomes in patients with dyslipidemia in Jordan.

Total cholesterol, LDL, and total cholesterol/HDL were significantly lower among the high adherent participants. Those who reported low or moderate adherence represented the majority of patients with uncontrolled dyslipidemia.

Our study shows that patients with a higher medication necessity score had a higher level of adherence, while those with greater medication concerns scores had lower adherence rates. Patient beliefs about their medications are essential determinants of whether or not to take medication as prescribed [29], and negative beliefs were found to be strong predictors of medication non-adherence [30]. The current study participants demonstrated negative beliefs about medications with greater medication concerns than medication necessity, which could justify the significant association between both medication necessity and concerns with adherence in the present study. A meta-analysis reported that beliefs about medications, including the necessity for and concerns about the prescribed medications, are essential factors to consider when investigating medication non-adherence [31]. Patients who believe that medication is necessary for improving health outcomes are more likely to adhere to their medication. In a cohort study of 1413 patients recently initiated on statin, results showed that patients who believed that statin was unnecessary to control their dyslipidemia were more likely to discontinue statin therapy [32]. In the same study, it was concluded that pharmacy records alone are insufficient to assess adherence; we should rather further investigate the reasons behind patients’ non-adherence. Patients with strong concerns about medications’ side effects, dependence, and disruption of daily activities are more likely to be non-adherent to the prescribed medications [31]. Consistent with this finding, previous studies reported that patients who were concerned about the potential side effects of their prescribed medications were found to be more likely to be non-adherent to their medications among patients with hypertension [21,33,34,35,36], diabetes [20], metabolic syndrome [37], and COPD [36]. A previous study found that the most common reason behind statin discontinuance were concerns about side effects, represented as muscle aches and gastrointestinal disorders [38]. Our results show that patients on no statin or moderate-intensity statin therapy showed high adherence levels compared to patients on a high-intensity statin. The significant association between increased concerns about side effects of the prescribed medications and adherence rates in the present study may justify the association between receiving a high-intensity statin and low adherence rates among the study participants. Therefore, choosing medications with lower side effects, addressing potential side effects, and offering guidance on mitigating or dealing with side effects can have the potential to help clinical pharmacists overcome the barrier of patients’ concerns about side effects.

The current study revealed a significant association between the increased number of prescribed medications and medication non-adherence among patients with dyslipidemia. This finding could justify the lower adherence rate reported by patients who received statin with fibrate compared with those who received statin monotherapy in the present study. This is consistent with the results of previous studies [39,40,41,42,43,44,45,46,47] where multiple regression analysis showed that the number of prescribed medications was found to be a significant and independent predictor of self-reported non-adherence. A previous study showed that the complexity and frequency of the therapeutic regimen are essential determinants of medication adherence in patients with diabetes and dyslipidemia [19]. In addition, a retrospective study showed that medication adherence was significantly higher in patients receiving a single-tablet lipid-lowering therapy than in patients receiving multiple-tablet lipid-lowering therapy [17]. Another retrospective study was conducted on 8988 patients in the United States, where a significantly higher adherence was reported among patients on a combination of fixed-dose lipid-lowering tablets versus multi-tablet combination [48]. Recently, a meta-analysis was conducted to compare the effect of single-pill combination and free-combination treatment on adherence and clinical outcome in patients with hypertension, dyslipidemia, or both of these conditions. The meta-analysis reported better medication adherence and clinical outcome in patients on single-pill combination as compared to free-combination treatment [49].

Based on these results, consideration should be given to simplifying the prescription regimen by reducing the number of medications taken per day when designing therapeutic regimens to manage patients with dyslipidemia in Jordan.

Consistent with the findings of previous studies [28,48,50,51,52,53], the multiple logistic regression analysis showed a significant association between increased disease duration and medication non-adherence. A study conducted by Marinho et al. [54] also found that increased duration of disease in patients with type 2 diabetes in Brazil was associated with increased risk of medication non-adherence. Another research study showed that patients receiving statin had misconceived care period expectations, including a misunderstanding that care would be completed when cholesterol levels decreased [55]. These findings highlight that patients with chronic conditions such as dyslipidemia tend to discontinue treatment over time, which may negatively affect clinical results. For future intervention plans intended to improve patients’ health outcomes with dyslipidemia, clinical pharmacists should specifically target patients with prolonged dyslipidemia. The current study’s findings can help guide future pharmaceutical care intervention programs that aim to prevent disease complications and improve health outcomes among patients with dyslipidemia.

## 5. Study limitations

Despite analyzing a wide range of socio-demographic and clinical variables, cause–effect relationships cannot be established since this is a cross-sectional study. 

## 6. Conclusions

The current study revealed a low adherence rate to pharmacological therapy in patients with dyslipidemia in Jordan. This study demonstrates modifiable factors related to poor adherence in patients with dyslipidemia, such as beliefs regarding perceived risk, medication harms, treatment duration, and the number of medications. Clinical pharmacists should specifically target patients with prolonged duration of dyslipidemia, simplifying the prescription regimen by reducing the number of medications taken per day, choosing medications with a better safety profile, addressing potential side effects, and offering guidance on mitigating side effects. This may potentially improve their medication adherence and health-related outcomes for patients with dyslipidemia. The current study findings may help guide the development of future pharmaceutical care intervention programs aimed at preventing disease complications and improving health-related outcomes among patients with dyslipidemia.

## Figures and Tables

**Table 1:** Association between adherence level and different sample characteristics.

Variable	Category	Low Adherence (n = 75)	Moderate Adherence (n = 92)	High Adherence (n = 61)
		Frequency (%) or Mean (±SD)		
Age (Years) *		62.49(±10.77)	59.62(±10.70)	58.38(±10.06)
Gender	Male Female	39(33.1) 36(32.7)	52(44.1) 40(36.4)	27(22.9) 34(30.9)
Marital Status	Married Not married	71(34.8) 4(16.7)	79(38.7) 13(54.2)	54(26.5) 7(29.2)
Education Level †	Low education level High education level	53(34.6) 22(29.3)	60(39.2) 32(42.7)	40(26.1) 21(28.0)
Living Condition	Alone With others	6(33.3) 68(32.3)	7(±38.8) 90(±42.8)	5(±27.9) 52(±24.7)
Income Level ‡	Low High	70(35.5) 5(16.1)	72(36.5) 20(64.5)	55(27.9) 6(19.4)
Active Smoking	Yes No	25(31.6) 50(33.6)	35(44.3) 57(38.3)	19(24.1) 42(28.2)
Having Comorbid Conditions	Yes No	74(32.7) 1(50.0)	91(40.3) 1(50.0)	61(27.0) 0(0.0)
ASCVD	Yes No	50(36.2) 25(27.8)	58(42.0) 34(37.8)	30(21.7) 31(34.4)
Hypertension	Yes No	58(35.8) 17(25.8)	64(39.5) 28(42.4)	40(24.7) 21(31.8)
Diabetes	Yes No Pre-DM	36(36.4) 34(30.4) 5(29.4)	39(39.4) 44(39.3) 9(52.9)	24(24.2) 34(30.4) 3(17.6)
Drugs **	No statin Moderate intensity statin Statin and fibrate High intensity statin	0(0.0) 15(14.0) 16(44.4) 44(67.7)	3(15.0) 49(45.8) 20(55.6) 20(30.8)	17(85) 43(40.2) 0(0.0) 1(1.5)
Controlled lipid profile **	Controlled # Uncontrolled	17(19.1) 58(41.7)	36(40.4) 56(40.3)	36(40.4) 25(18.0)
Duration Of Dyslipidemia **		20.15(±8.35)	11.97(±4.58)	3.38(±1.96)
BMI $		30.25(±6.81)	27.87(±4.92)	29.07(±5.88)
Number Of Chronic Condition **		3.04(±0.99)	2.99(±1.12)	2.59(±0.92)
Number Of Medication **		8.60(±1.66)	6.01(±2.09)	3.48(±1.85)
Total cholesterol **		264.12(±76.42)	269.39(±99.56)	181.96(±77.84)
Triglyceride *		149.40(±52.02)	137.28(±46.03)	129.74(±46.23)
HDL		45.73(±8.82)	48.30(±8.63)	48.41(±7.31)
LDL **		188.51(±72.12)	193.63(±98.18)	107.61(±74.05)
Total cholesterol/HDL **		6.01(±2.07)	5.89(±2.75)	3.91(±1.95)
Triglyceride/HDL **		3.42(±1.40)	2.95(±1.16)	2.82(±1.40)
Necessity ¶ **		10.60(±3.35)	13.26(±3.62)	15.97(±4.70)
Concern § **		19.75(±3.01)	17.13(±3.45)	12.93(±4.42)

† Educational level was classified as follows: high for patients who completed university level or more and low for patients who had primary, secondary, or high school. ‡ Average monthly income was classified as low (<1000 JOD) and high for (≥1000 JOD). $ BMI (body max index) was classified as normal (19.9–24.9) and high (≥ 25). ¶ The mean of the participants’ responses to the necessity items in the Beliefs about Medicines Questionnaire. § The mean of the participants’ responses to the concerns items in the Beliefs about Medicines Questionnaire. * Significant at *p*-value < 0.05, ** Significant at *p*-value < 0.01. # Patients were categorized as controlled and uncontrolled dyslipidemia groups based on lipid profile records. Patients who were not achieving their LDL-C, TG, or HDL-C target levels were considered to have an uncontrolled lipid profile.

**Table 2:** Beliefs about medication questionnaire (BMQ) items.

Necessity Items	Mean of Each Item (SD)
My medicine to lower my cholesterol protects me from becoming sick	2.32 (1.10)
My health depended on medicine to lower my cholesterol	2.53 (1.12)
Lowering my cholesterol requires medication	2.46 (1.17)
My life would have been impossible without medications to lower my cholesterol	3.04 (1.23)
Without medicine to lower my cholesterol, I may have become very ill	2.79 (1.19)
**Concerns items**	
I sometimes worried about becoming too dependent on medicine to lower my cholesterol	3.36 (1.06)
My medicine to lower my cholesterol disrupted my life	3.50 (0.99)
I sometimes worried about the long-term effects of medicine to lower my cholesterol	3.32 (1.06)
Having to take medicine to lower my cholesterol worried me	3.37 (1.05)
My medicine to lower my cholesterol was a mystery to me	3.32 (1.05)

**Table 3:** Stepwise ordinal regression indicating variables associated with adherence level.

Variables	Adjusted Coefficient	*p*-Value	95% Confidence Interval	
			Lower Bound	Upper Bound
Necessity	0.43	<0.01	0.27	0.58
Concerns	−0.41	<0.01	−0.57	‒0.25
Duration of dyslipidemia in years	−0.22	<0.01	−0.31	−0.13
Number of medications for all conditions	−0.64	<0.01	−0.92	‒0.36
Statins intake (Reference: High-intensity statins) No statin Moderate intensity statin Statin and fibrate	4.01 2.34 2.04	<0.01 <0.01 <0.01	1.65 1.08 0.64	6.37 3.593.44

## Data Availability

Data is contained within the article.
